# A Systematic Literature Review and Bibliometric Analysis of Blockchain Technology for Food Security

**DOI:** 10.3390/foods13223607

**Published:** 2024-11-11

**Authors:** Balan Sundarakani, Anis Ghouse

**Affiliations:** School of Business, University of Wollongong in Dubai, Knowledge Park, Dubai P.O. Box 20183, United Arab Emirates; asg972@uowmail.edu.au

**Keywords:** blockchain, food security, food trust, food supply chain, literature review

## Abstract

The United Arab Emirates (UAE) faces obstacles in guaranteeing food security because of its desert climate, restricted arable land, and significant reliance on food imports. Establishing a robust and transparent food supply chain is crucial. This study investigates the crucial functions of blockchain technology in protecting and improving food security in the UAE. Using bibliographic and co-citation network analyses, this study examines 143 research articles that provide a thorough review of the current status of blockchain technology in relation to food security. We examine the interrelationships among studies, highlighting significant themes and identifying three emerging food security patterns in the incorporation of blockchain into the food security domain. This study enhances the understanding of how blockchain technology can transform the food security dimensions of availability, accessibility, utilization, and stability in the UAE and worldwide.

## 1. Introduction

In the age of technological advancement and increasing global concern for food security, innovative technologies have sparked a paradigm change in how we address nations’ food security challenges. Food security is of utmost importance, as the ever-growing global population requires efficient food production and distribution to ensure that everyone has access to an adequate and reliable food supply [1]. Food distribution within the supply chain must be managed effectively to avoid food waste and loss during production, procurement, storage, and distribution processes. The effective design and management of the food supply chain contribute to food availability, accessibility, and security [2,3]. Innovative technologies such as blockchain are distinguished by their originality, disruptive potential, and capacity to bring about substantial changes that reshape how we think about trust, security, and transparency in the digital age.

**Figure 1:**
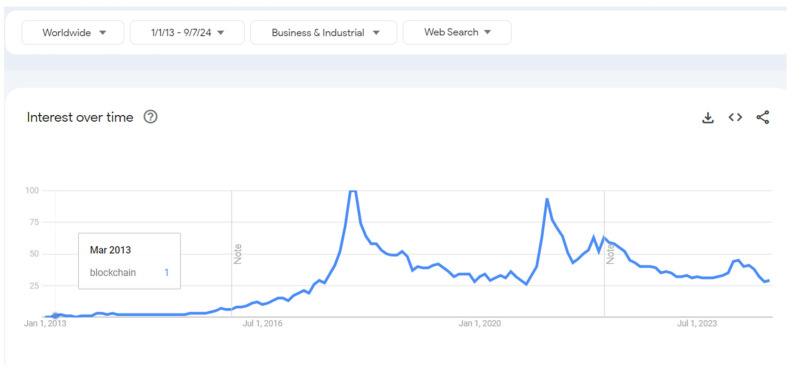
A Google trend search of the past 12 years for blockchain in the context of business and industry [source: data sourced from Google and created by the authors].

Fundamentally, a blockchain is a decentralized, distributed ledger that documents transactions across a network of computers and is distinguished by its immutability and encrypted security, which render it virtually impervious to tampering and fraud. Although blockchain as a decentralized ledger was created by Nakamoto in 2008 [3], the application of blockchain in business and industry context was first registered in March 2013, as shown in Figure 1. While blockchain research has grown since then in many industry verticals including the automobile, real estate, and banking industries, the application of blockchain in food security and the agri-food supply chain industry has been minimal and nascent at present.

Blockchain provides an immutable and transparent record of the food supply chain through its decentralized ledger architecture. This transparency allows the tracing of food origins and journeys, ensuring authenticity and safety. Businesses integrate blockchain technology into their food supply chains to substantially enhance food security.

The IBM Food Trust is a revolutionary platform that employs blockchain technology to improve the transparency, traceability, and safety of food supply chains. This innovative solution, developed by IBM, addresses food security challenges. The IBM Food Trust combines supply chain modules with blockchain fundamental functions to deliver business value to the food ecosystem by combining governance, standards, and interoperability with technology [4]. Antonello Produce, a family-owned and operated business that provides wholesale, marketing, and distribution services for fresh produce to grocers, restaurants, and consumers throughout Australia’s southeastern region, uses the IBM Food Trust to streamline product traceability from seeds to store shelves. The Antonello Produce Whole program primarily relies on the IBM Food Trust, where the company only needs a batch number or an invoice number to offer a comprehensive history for that product (“Safe, sustainable, satisfying—what’s on your plate matters”, n.d.). Similarly, several Norwegian seafood companies, including Kvarøy Arctic and BioMar, have joined the blockchain network, providing information on seafood origin and quality and its feed quality. These companies use blockchain technology to generate a “single version of the truth” concerning supply chain events, such as product origins and preparation. They also use private blockchain networks to record catch location and time, supply chain events like shipping updates and customs clearance, and even temperature, which can be shared with permission parties to improve the quality of Norway’s exports [5]. As blockchain technology evolves, food traceability has transformed, making it a promising solution for addressing food security challenges.

**Figure 2:**
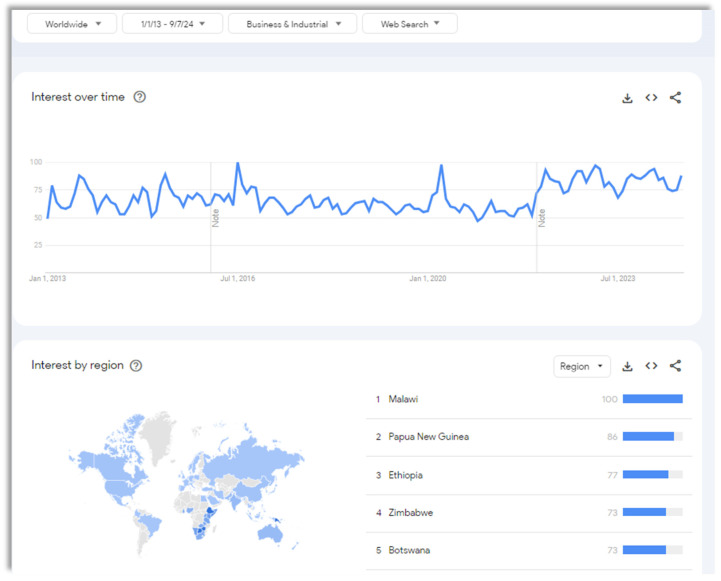
A Google trend search of the past 12 years for food security [source: data sourced from Google and created by the authors].

The concept of food security has changed since its initial introduction into policy discussions in the early 1970s. Over the course of subsequent years, there has been a growing consensus regarding the fundamental components of food security, commonly recognized as the four essential pillars: availability, access, utilization, and stability [6]. Food security is a critical aspect of a nation’s wellbeing, representing its ability to ensure that its population has consistent access to safe, nutritious, and sufficient food to lead a healthy, active life. It is about having adequate food and ensuring that it is affordable and culturally acceptable. Figure 2 depicts the significance of food security and the impact of it worldwide for the last 12 years. As this sets the ground for food security investigation further, the role of blockchain in food security remains an under-researched topic that has merit for both practitioners and academics. Therefore, this research is an early attempt to fill this gap.

Globally, the goal of providing food security for a nation is complex and fraught with several obstacles. These include a growing population, the escalating effects of climate change, resource scarcity, income inequality, economic disruptions, conflicts, food waste, inadequate infrastructure, and a lack of education. Blockchain technology can address several challenges by enhancing the transparency and traceability of the food supply chain. The MENA region includes a broad spectrum of nations with diverse economic and developmental statuses. This diversity extends from low-income and least-developed countries such as Sudan, Mauritania, and Yemen to high-income, well-developed, oil-exporting nations such as the UAE, Qatar, and Saudi Arabia [7]. Despite significant variation in economic profiles across the MENA region, shared climate-related hurdles pose a unifying factor in the struggle to ensure food security (“Breaking the Cycle”, [8]) The United Arab Emirates (UAE) is considered food secure because of its economic security and ability to purchase food in the international market at higher prices. The UAE’s glaring challenge, which distinguishes it from many other food-secure nations, is climate change. As an arid nation devoid of water and arable land, the United Arab Emirates faces unique food security challenges. With an expanding population and rising consumption demands, the UAE must confront this obstacle head-on because of its inability to produce food; it imports approximately 80–90% of its food. Nevertheless, food security remains a long-term concern due to production and importation supply challenges [9]. The UAE imports food, resulting in net imports, rendering the UAE susceptible to food price fluctuations and supply disruptions. Blockchain technology can help the UAE address food security through the traceability of food products throughout the supply chain, identify and prevent food fraud, ensure food safety and quality, and reduce food waste by providing insights into demand and supply patterns. As the UAE endeavors to achieve food security, blockchain technology has emerged as a pivotal solution. The bibliometric analysis conducted in this study addressed two crucial research questions (RQs).

RQ 1: How has blockchain as a technology been utilized in the context of food security?

RQ 2: How does blockchain as a technology contribute to ensuring traceability, transparency, and security in the food supply chain?

The remainder of this paper is organized as follows. Section 2 presents a literature review. Section 3 describes the research methodology. Section 4 discusses the review using bibliometric analysis. Based on the evaluation results, Section 5 presents the network analysis. Section 6 concludes with limitations and suggested areas for future research.

## 2. Literature Review

This section discusses the multifaceted dimensions of food security, with a particular focus on exploring the intricacies of ensuring food security in the UAE while considering the country’s unique challenges and opportunities and the role of blockchain technology in bolstering food security within the UAE. It also examines how this innovative technology can revolutionize supply chain management, enhance traceability, and promote sustainability.

### 2.1. Food Security

According to the United Nations, the global population is expected to reach 9.7 billion by 2050 (United Nations [10]). This exponential growth imposes a tremendous strain on global food systems (“Food Loss and Waste GCC Solutions for A Global Challenge” [11]). This rapid and unprecedented population growth has elevated the significance of ensuring food security, which is widely recognized as a critical concern on an international scale, given that it signifies the degree of self-reliance of a nation and the welfare of its people. According to the 1996 World Food Summit, food security is defined as having physical and economic access to adequate, safe, and nutritious food that meets the dietary needs and food choices necessary for an active and healthy life. Food security encompasses the following four dimensions. The first is the physical availability of food, which pertains to the supply side of the equation and relies on factors such as food production, stock levels, and net trade. The second dimension, economic and physical access to food, recognizes that a sufficient national or international food supply is needed to guarantee food security at the household level. This dimension requires a focus on income, expenditure, markets, and prices to ensure that individuals have access to the food they need. The third dimension, food utilization, emphasizes how the human body effectively utilizes consumed nutrients. This involves good care and feeding practices, food preparation, dietary diversity, and equitable food distribution within households. The fourth dimension, stability, underscores the need for consistency over time in the other three dimensions. To achieve food security, all four dimensions must be addressed concurrently to ensure that food is physically available, economically accessible, nutritionally utilized, and stable over time. The food security of a nation is impacted by its food supply chain, which consists of entities and processes that include production, transportation, manufacturing, retailing, consumption, and waste management (“What is Food Security?” [12]). According to comprehensive research from 2011 conducted by the United Nations and Food and Agriculture Organization (FAO), approximately one-third of all food produced worldwide for human consumption is lost or wasted each year [13].

This represents a staggering 1.3 billion tons of food and additionally, the economic ramifications are substantial, with an estimated USD 1 trillion worth of food wasted annually (“Food Loss and Waste GCC Solutions For A Global Challenge.pdf” [14]). According to the 2019 Food and Agriculture Organization (FAO update [11]), 14% of this loss occurs before the food reaches retailers; a predominant factor contributing to this significant pre-retail food loss is inefficiency within the food supply chain. Throughout the entire journey from production and harvesting to processing, distribution, and consumption, opportunities for food loss manifest at various stages in the supply chain (“Food Loss and Food Waste|Policy Support and Governance Gateway|Food and Agriculture Organization of the United Nations|Policy Support and Governance|Food and Agriculture Organization of the United Nations”, n.d.) (“Food Loss and Waste GCC Solutions For A Global Challenge.pdf” [14]). Food loss in the food supply chain has significant implications for national food security. The digitization era has brought about a huge transformation in the approach to food security, as technologies such as blockchain enable creative solutions that help improve transparency, traceability, and efficiency in the supply chain, ultimately improving food security [15].

### 2.2. Food Security in the UAE

The UAE has developed an ambitious food security strategy intended to position itself as the leading nation on the Global Food Security Index (GFSI) by 2051. This ambitious endeavor underscores the nation’s commitment to ensuring a stable, sustainable, and resilient food supply for its growing population (“Food Loss and Waste GCC Solutions For A Global Challenge.pdf” [14]. With a determined focus to be self-reliant, the UAE is actively pursuing the integration of cutting-edge technologies and innovative cultivation practices, such as vertical farming, hydroponics, organic farming, and controlled-environment agriculture, but due to its limited arable land, increasing climate challenges, and acute water scarcity, the country still relies heavily on food imports to meet 90% of its food demands of the population (“A Guide to Food Security in the UAE.pdf” [7,15].) The significance of prioritizing food security policies came to the forefront in 2007, as escalating global food prices presented the UAE with challenges in securing fundamental commodities (“A Guide to Food Security in the UAE.pdf” [7]). The UAE exhibits a notable reliance on imported food commodities, particularly prepared dairy, food, fresh fruit, poultry, tree nuts, and beef products, which are supplied by the European Union, India, Brazil, the United States, and Saudi Arabia, which are the principal suppliers. In addition to this, the UAE also maintains a very comprehensive system of inspections and monitoring to ensure compliance with food quality and food safety regulations (“A Guide to Food Security in the UAE.pdf” [7]) (“Food Loss and Waste GCC Solutions For A Global Challenge.pdf” [16]. The UAE’s food import bill is expected to be around USD 14 billion in 2020, up from roughly USD 10 billion a decade ago [17]. The UAE imported USD 12.5 billion in consumer-oriented products in 2022, accounting for 59% of total Agricultural imports [17]. The Strategy for National Food Security in 2051 establishes the components of the national food basket, comprising 24 primary food categories, according to three standards: understanding of the magnitude of domestic consumption of the most critical products, the capabilities of production and processing, and nutritional requirements (“A Guide to Food Security in the UAE.pdf” [7]).

According to UAE government statistics, the nation has 568 food and beverage processors. These firms generate 5.96 million metric tonnes (MT) of food and beverage products annually, with staple food items accounting for 2.3 MT. This sector also primarily relies on imported commodities and ingredients due to low domestic agricultural production and is important in the government’s attempts to improve food security and basic commodity self-sufficiency [18]. Due to its high dependency on food imports, the UAE places significant importance on maintaining national food reserves to ensure a stable and secure food supply. These reserves serve as strategic buffers against disruptions in food availability or price volatility caused by disasters, global market swings, and geopolitical events. Strategic food security reserves preserve crucial food commodities and staples, such as rice, wheat, sugar, pulses, edible oils, and other commodities that are critical for supporting the population’s basic needs. These reserves are managed and monitored by specialized bodies that oversee stockpile levels, guarantee suitable storage conditions, and maintain the quality and safety of stockpiled food supplies. To successfully manage and monitor the reserves, regular inspections, quality assessments, and inventory management systems are used (“A Guide to Food Security in the UAE.pdf” [7]). Situated at the crossroads of Europe, Asia, and Africa, the UAE holds a significant and strategic global position as a commercial hub for trade and a cornerstone of the global movement of goods. Its state-of-the-art infrastructure, including world-class ports and airports, allows for the seamless transit of products which has a substantial impact on both regional and global food supply chains in efforts to ensure food security (“Food Loss and Waste GCC Solutions For A Global Challenge.pdf” [16]) (“A Guide to Food Security in the UAE.pdf” [7]) [19]. Enhancing food chain transparency is vital for food security, especially in light of innovative agricultural practices, the maintenance of national food reserves, and substantial food imports.

### 2.3. Blockchain

In the face of global challenges concerning food security, technology has emerged as a crucial collaborator, and blockchain plays a significant role in this transformative endeavor. Blockchain technology, which has gained prominence in the context of cryptocurrencies, is currently demonstrating its transformative potential in food security by addressing various food security challenges such as mitigating risks, minimizing fraud, optimizing logistics, and offering an immutable and real-time record of transactions [20,21].

#### 2.3.1. Blockchain Technology

**Figure 3:**
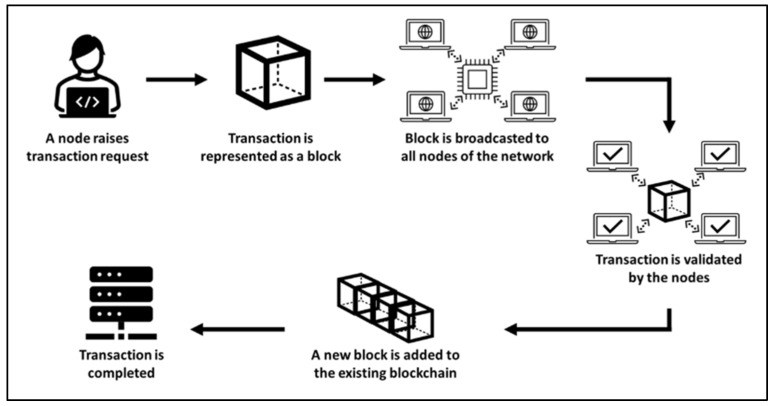
How blockchain as technology works, and its stages [source: authors’ diagram].

Blockchain technology functions as a decentralized and distributed ledger system that operates within a peer-to-peer network. When a transaction is disseminated in a blockchain network, every occurrence is recorded as a singular “block” of data, forming a transparent and immutable ledger [22]. These real-time recorded transactions provide a transparent view of the movement of assets, such as tangible entities in the form of products, or intangible digital entities, such as intellectual property [23]. The versatility of a data block is a remarkable feature that enables it to accommodate large volumes of data. For details, refer to Figure 3.

The block comprehensively records the precise details of the transaction, encompassing information on the initiator (“who”) and the nature of the exchange (“what”). The data block also preserves the intricate narrative of “when” and “where” the event transpired. Consequently, each block serves as proof of the complex and all-encompassing characteristics of the transaction, fostering confidence, ensuring protection, and promoting responsibility in the digital domain [24,25]. Each block is intricately connected to the preceding block with its hash, and subsequent blocks utilize the previous block’s hash to compute their hash, creating an unbroken and unchangeable chain of data. This continuous and immutable data chain operates as a ledger that methodically documents an asset’s path during ownership transfers or movements between locations. The cryptographic linkage established by these hashes ensures the immutability of past transactions, preventing any blocks from being altered or new blocks from being inserted between two existing blocks. Consensus mechanisms, such as Proof of Work or Proof of Stake, facilitate agreements among nodes regarding the validity and order of transactions. This decentralized consensus prevents a single entity from controlling the entire network. The result is a transparent, tamper-resistant, and highly secure system that fosters trust and accountability in various applications ranging from cryptocurrency transactions to smart contracts [25].

#### 2.3.2. Types of Blockchain Network

A wide range of networks characterize blockchain technology ecosystems, each tailored to meet specific organizational, security, and operational needs. There are several types of blockchain networks, namely, public, private, permissioned, and consortium networks [26]. We examined the distinctive attributes of each category to uncover the qualities that set them apart. A deep understanding of the intricacies of the different types of blockchain networks is crucial for effectively navigating the ever-changing world of ledger technology [27]. This knowledge enables businesses and organizations to make well-informed decisions that are customized to their unique requirements and goals.

##### Public Blockchain Networks

Public blockchain networks, such as Bitcoin, function based on a decentralized philosophy that allows open participation by all individuals [28]. Although the inclusive aspect of the system promotes openness, it also has limitations such as the requirement for substantial processing capacity, restricted transaction privacy, and possible security flaws [29]. These factors are particularly relevant when assessing the appropriateness of public blockchains for commercial applications. The balance between the openness and difficulties presented by computing requirements and privacy issues must be carefully considered [26].

##### Private Blockchain Networks

In a private blockchain network, such as its public version, decentralization is upheld through peer-to-peer networks [30]. Nevertheless, the responsibility for governance is transferred to the sole entity that supervises participation, monitors the consensus procedure, and administers the shared ledger [30,31]. Centralized control fosters trust and confidence among participants, which is a critical determinant that depends on a particular use case [32,33]. Private blockchains can function within the confines of a corporate firewall or be hosted on-premise, thereby customizing the technology to suit the particular requirements of a business [33].

##### Permissioned Blockchain Networks

Businesses opting for private blockchains often establish permissioned blockchain networks. Public blockchains can also adopt permission models [34]. In a permissioned network, participation and transaction access are restricted, requiring participants to obtain invitations or permissions before joining the network [28]. This controlled approach adds an additional layer of security and is particularly relevant in scenarios where selective participation is crucial.

##### Consortium Blockchains

Consortium blockchains enhance the upkeep of blockchains by incorporating collaborative aspects. Various organizations are collectively obligated to maintain the blockchain and determine the eligibility for transaction submissions and data access. The shared governance model is particularly suitable for business scenarios in which all participating entities require permitted access and collectively bear the responsibility of maintaining the integrity of the blockchain [28]. Consortium blockchains offer a favorable equilibrium between decentralization and regulated cooperation.

#### 2.3.3. Advantages of Blockchain Technology

##### Enhanced Security

Ensuring the security and confidentiality of data is of the utmost importance, and blockchain technology provides a revolutionary method for protecting vital information. Blockchain is a robust deterrent against fraud and unlawful activity that creates permanent and securely encrypted records [34]. Blockchain effectively addresses privacy issues by anonymizing personal data and implementing strict permissions to regulate access [35]. In contrast to traditional single-server storage, a blockchain employs a decentralized network of computers to distribute information. This approach dramatically enhances security by making it arduous for hackers to obtain illegal access to and examine data [36].

##### Traceability

Blockchain offers significant benefits for increasing transparency [37]. In contrast to conventional systems, where each business manages its database, blockchain utilizes a distributed ledger to guarantee the consistent recording of transactions and data across numerous locations [14,38]. When all parties are given authorized access, they can simultaneously access the same information, which promotes complete openness. The unchangeability of documented transactions, along with accurate time and date records, enables members to examine the complete history of each transaction, effectively eliminating the chances of fraudulent operations [14]. The transparent and decentralized nature of blockchain fundamentally transforms the storage and sharing of information among network participants [39].

##### Increased Efficiency and Speed

Conventional paper-intensive procedures are characterized by time-consuming workflows, susceptibility to human error, and frequent reliance on third-party mediation. Blockchain integration streamlines these processes, resulting in faster and more efficient transactions [40]. Documentation and transaction details can be stored securely in blockchain, eliminating the need for paper exchange. The cumbersome task of reconciling multiple ledgers has become obsolete, paving the way for significantly expedited clearing and settlement processes [41,42]. Through the efficiency gains offered by blockchain technology, businesses can overcome the limitations of traditional paper-based systems and embrace a streamlined, error-resistant approach to transaction management [43,44].

##### Automation

Automation reaches a new level by implementing “smart contracts”, enhancing efficiency and expediting processes. These intelligent contracts automatically initiate the next step in a transaction or process once prespecified conditions are met. This automation significantly reduces the need for human intervention and minimizes the reliance on third parties to verify contract compliance [41]. For example, smart contracts enable seamless and swift claim settlements in sectors such as insurance. When a customer fulfils all the necessary documentation requirements, the smart contract triggers automatic settlement and payment, streamlining the entire process and minimizing delays [36].

##### Smart Contracts

Smart contracts operate based on straightforward “if/when…then…” conditions encoded into the blockchain. Once predetermined conditions are met and verified, a network of computers executes specified actions, ranging from releasing funds to registering a vehicle, sending notifications, or issuing a ticket [44]. The completion of a transaction updates the blockchain, ensuring its immutability, and only authorized parties can access the outcomes.

Participants can include numerous conditions to ensure satisfactory task completion within the smart contract. To establish these terms, participants must decide on the representation of transactions and data on the blockchain, agree on the governing “if/when…then…” rules, anticipate exceptions, and outline a dispute resolution framework. This meticulous process ensures clarity and consensus among participants in a smart contract.

## 3. Research Methodology

**Figure 4:**
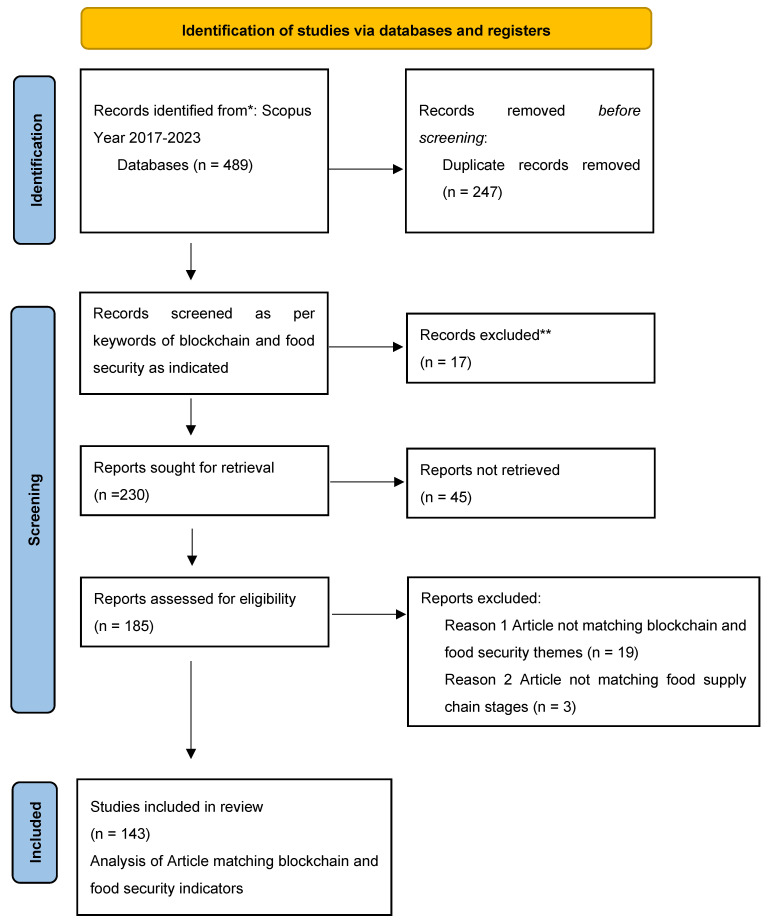
A review methodology flow chart as per PRISMA guidelines 2020 [source: authors’ work adopted from [47]].

A literature review is a fundamental component of a research project, providing beneficial insights for exploring new areas and shaping the direction of future research. Research gaps were identified by carefully analyzing and assessing the pertinent literature, which served as a basis for strengthening the study. This systematic literature review used an iterative approach, including identifying appropriate search keywords, examining relevant materials, and analyzing the findings [45,46]. Within the scope of this study, the primary sources of publication were in the domains of blockchain in food security and food supply chains. The articles were obtained from the Scopus database, which is well known for its extensive collection of peer-reviewed literature. The review process followed a six-step methodology, as illustrated in Figure 4, depicted in the flowchart.

### 3.1. Selection of Database

The researchers developed search techniques that leverage pertinent data sources. Scopus was selected as the preferred database to ensure the availability of a wide range of studies for this evaluation. Scopus is a widely used abstract and citation database that includes many articles and abstracts from peer-reviewed journals in various fields, such as technology, agriculture, management, and supply chains.

### 3.2. Collection of Articles

The literature review comprised of papers published between 2017 and 2023. After the initial search, 489 articles were identified. To establish a solid basis for this research, the search strategy eliminated editorial remarks, book chapters, and doctoral theses as sources and focused solely on scholarly sources. Further improvements in accordance with the defined criteria led to a decrease in the number of articles to 247.

### 3.3. Keyword Selection

While conducting their research, the authors exclusively cited the most noteworthy and essential research articles. The authors utilized specific keywords, such as “blockchain”, “hyperledger”, “distributed ledger technology”, “agriculture supply chain”, “food supply chain”, and “agriculture” as part of their search strategy. The inclusion of these keywords also outlines the boundaries for bibliometric analysis within the context of the ongoing study.

### 3.4. Filtering

To improve accuracy, incomplete bibliographic data points in the redundant and duplicate papers were eliminated from the evaluation, thus leading to 143 papers taken for full bibliometric and network analysis. This process of filtering is based on the guidelines of the PRISMA standard of literature reviews, and thus follows compliance with the PRISMA guidelines [47], as shown in Figure 4. These processes has been explained in this Section 3.1, Section 3.2, Section 3.3 and Section 3.4 earlier.

### 3.5. Data Statistics

**Figure 5:**
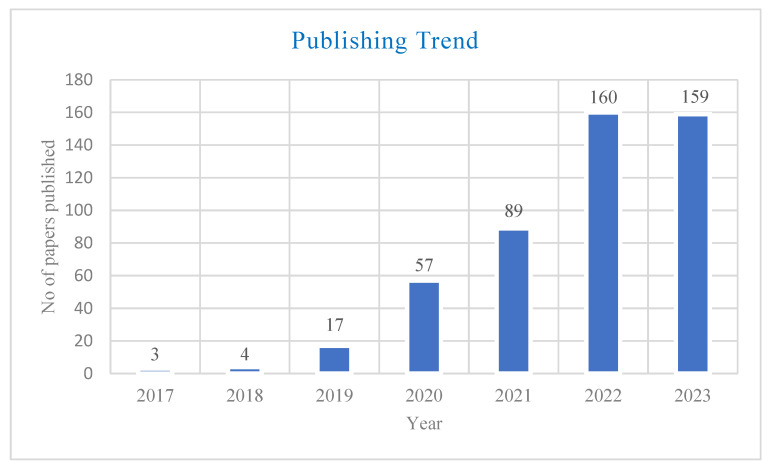
Publishing trends in blockchain and food security [source: authors’ own work].

The quantity of published papers on the role of blockchain technology demonstrated a substantial upward trend over the years, indicating a noticeable rise. The number of papers increased by 33.33% from 2017 to 2018, and from three to four. In 2019, there was a significant jump with a 325% increase of 17 studies. In 2020, the growing trend persisted, and a significant surge of 235.29% was witnessed, resulting in 57 publications. In 2021, there was a strong and steady increase, with a significant growth rate of 56.14%, resulting in 89 publications. By 2022, there is a significant increase of 79.78%, resulting in 160 published publications. In 2023, there is a marginal decline of 0.63% (159 papers), suggesting a stabilization in the growth trend. In general, these percentages highlighted the changing and growing nature of studies on this topic over time. For details, refer to Figure 5.

## 4. Bibliometric Analysis and Discussion

Bibliometric analysis is a quantitative method used to assess many elements of scholarly publications and usually focuses on a specific topic or profession. This approach involves a systematic analysis of bibliographic information such as the number of times a journal is cited, patterns of authorship, and trends in publications. The goal is to derive valuable insights into the organization and influence of scientific literature. Bibliometric analysis is commonly used in academia and research organizations to evaluate the impact of research, identify trends, and inform strategic decision-making. Researchers, institutions, and policymakers use bibliometric indicators to assess the research output and its effects, distribute resources, and identify possible partners. Bibliometric analysis is crucial for understanding the dynamics of scholarly communication and advancing scientific knowledge in specific disciplines. Previous research has used various software tools with varying capabilities and restrictions to conduct bibliometric analyses. Considering these limitations, the present study utilized R for the first statistical analysis and data preprocessing step, thereby facilitating the subsequent network analysis using VOS Viewer.

### 4.1. Publishing Outlets

The prominent publication platforms in this study have various sources that contribute to the distribution of research articles. *Sustainability* (Switzerland) is notable for its 28 pieces, highlighting a strong focus on sustainable practices and multidisciplinary research. The journals *IEEE Access* and *Journal of Cleaner Production* have 24 and 22 papers, respectively. These articles indicate a strong emphasis on technological progress and environmentally friendly manufacturing. The journals *Foods* and *Sensors* each have 11 articles, which investigate advancements in the food sector and sensor technologies. The journal *Computers and Electronics in Agriculture* features seven essays exploring the convergence of technology and agricultural practices. *The International Journal of Advanced Computer Science and Applications* contains seven articles, while *Operations Management Research* includes six papers. These results suggest the need for a wide range of research in advanced computing and operational management. Both *Agriculture* (Switzerland) and *IEEE Transactions on Engineering Management* have five papers each, making a total of 10 articles that contribute to the literature on agricultural sciences and engineering management. These publishing platforms highlight the wide range of ways in which research findings are shared across different fields of study. Table 1 presents the results by journal.

### 4.2. Keyword Occurrences

**Figure 6:**
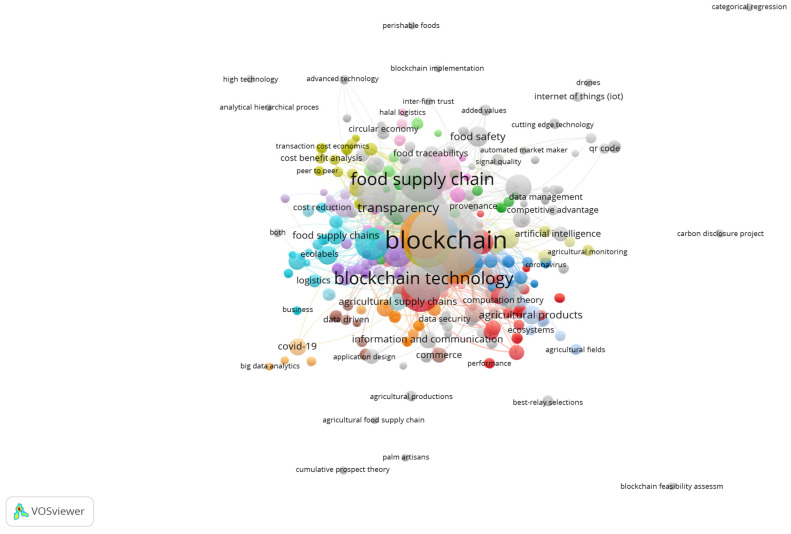
Keyword occurrences [source: authors’ own work].

Keyword occurrences highlight the thematic focus and emphasis within the research articles, reflecting current trends and priorities in academic discourse. Notably, “blockchain” emerges as the predominant keyword with 269 occurrences, suggesting a significant interest in exploring the applications and implications of this technology. The variations “blockchain” and “blockchain technology” further underscore the attention given to the nuanced aspects of blockchain. “Food supply” and “supply chains” follow closely, with 137 and 116 occurrences, respectively, indicating a substantial body of research dedicated to understanding and improving food supply chains. The prevalence of such keywords as “internet of things”, “agriculture”, and “supply chain management” points toward the intersection of technology, agriculture, and logistics. The inclusion of such terms as “agricultural robots”, “food safety”, and “sustainable development” underscores the research community’s commitment to advancing agricultural technologies while addressing critical issues related to safety and sustainability. The frequency of keywords such as “smart agriculture”, “network security”, and “smart contract” reflects a growing interest in integrating innovative technologies and ensuring secure digital practices within the agricultural domain. The distribution of keywords provided valuable insights into the current research landscape, showcasing the multidimensional nature of investigations in agriculture, technology, and supply chain management. Refer to Table 2 and Figure 6 for details on keyword occurrences.

### 4.3. Thematic Analysis Using Strategic Diagram

**Figure 7:**
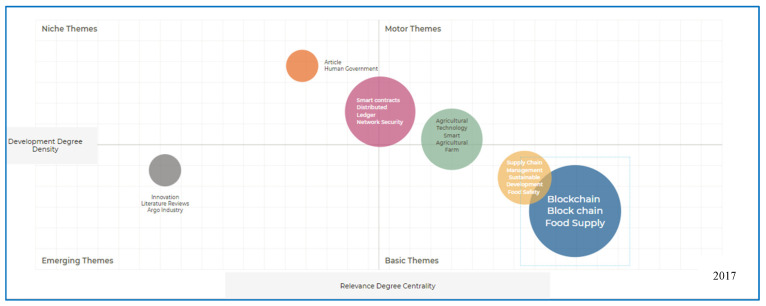
Strategic diagram: 2017–present [source: authors’ own work].

**Figure 8:**
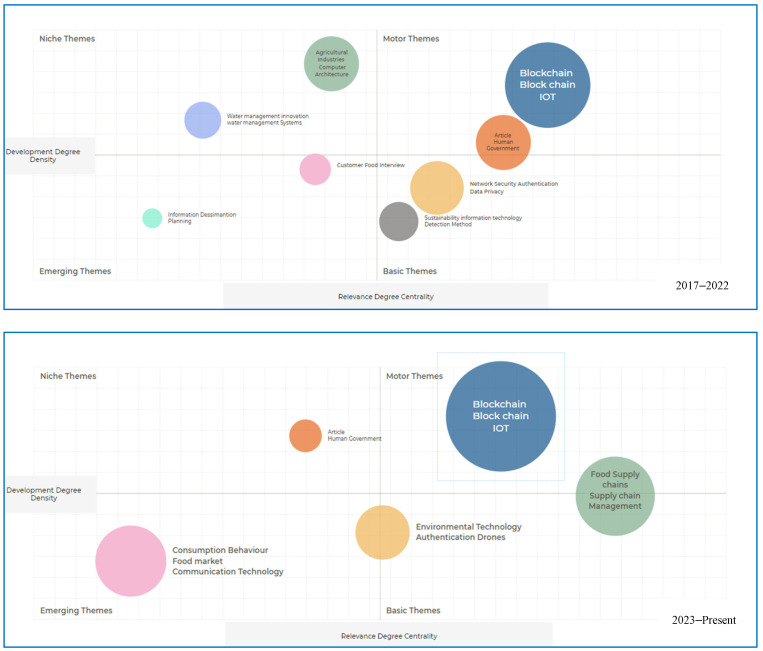
Strategic diagram: 2017–present [source: authors’ own work].

Thematic analysis is an essential component of bibliometric analysis as it contributes to thoroughly examining and understanding quantitative data. Thematic analysis involves the identification and classification of recurring themes or topics in a collection of academic literature. This approach enables researchers to identify the patterns, trends, and emerging areas within specific domains. Thematic analysis provides qualitative insights into bibliometric studies, which are primarily quantitative, by analyzing keywords, author-supplied terms, and other textual aspects. A comprehensive examination was conducted to identify emergent research trends pertaining to the use of blockchain technology to enhance food security. This study aims to quantify the co-occurrence of keywords in the indexed literature by employing co-word analysis. This methodology is crucial for identifying significant concepts and understanding commonly used terms within a discipline. It is impossible to overstate the importance of keywords in identifying emergent trends given that they function as linguistic indicators for research. In pursuit of additional categorization of these themes, scholars have employed strategic diagrams in accordance with the approach outlined by [48]. Strategic diagrams illustrate a two-dimensional representation, where the *y*-axis represents density and the *x*-axis represents centrality. The analysis of graphs categorized into four quadrants (emerging themes, basic themes, motor themes, and niche themes) serves as a valuable framework for understanding the evolution of concepts and technologies. Blockchain technology emerged as a basic theme in 2017, signifying its growing recognition and exploration beyond its initial applications. Over subsequent years, the graph illustrates notable progression, with blockchain transitioning into the motor theme quadrant by 2023. This shift reflects the technology’s maturation and widespread adoption across various industries, where specialized applications have become an integral component of solutions in the supply chain. The journey of the blockchain within these quadrants highlights its transformative impact and underscores the significance of tracking the development and integration of emerging technologies over time (see Figure 7 and Figure 8).

## 5. Network Analysis and Discussion

This study focused on a detailed analysis of a comprehensive network and a graphical investigation of selected data samples. The selected bibliographic dataset stored in the CSV format facilitates efficient data processing and manipulation, which provides a comprehensive foundation for citation and co-citation analysis. This study utilized the capabilities of the R 4.3.3 software and VOS Viewer, both of which are recognized as open-source tools with robust analytical features. Through the adept utilization of R software and VOS Viewer, this study aimed to explore and reveal the complex network interconnections and patterns within the scholarly domain by leveraging the open-source nature of these tools to enhance the transparency and accessibility of the analytical approach.

### 5.1. Citation Analysis

Citation analysis is a quantitative technique used in academic research to assess the impact and influence of scholarly publications by analyzing their citation patterns. Citation analysis offers valuable insights into the visibility and value of intellectual contributions by determining the frequency with which other researchers cite specific articles. This approach not only facilitates the evaluation of the influence of individual works but also enables scholars to identify significant publications and influential authors and develop patterns within a particular subject. In addition, citation analysis plays a role in developing citation networks, which expose the interconnections among the academic literature. Citation analysis is commonly employed in bibliometrics to assess research productivity, evaluate the scholarly influence of authors and journals, and gain a detailed understanding of the intellectual terrain across different academic fields.

Globally cited documents [Table 3] provide an exhaustive synopsis of the scholarly significance of a selected subset of publications by examining their citation impact and annual citation rates. An article authored by Dutta in 2020 [49] was at the top of the rankings, receiving 469 citations at an annual citation rate of 117.25. While study of [44] garnered a cumulative count of 463 citations, with an annual average of 115.75 recognitions. And [50] book received 385 citations in 2020, representing an annual average of 96.25 citations. This achievement record underscores consistent recognition by the authors. In 2019, Salah [51,52] received 372 citations, or 74.4 citations annually on average. In 2020, Behnke [53] contribution garnered 364 citations, at an average annual rate of 91. The 2018 paper authored by Leng Garnered received a cumulative sum of 295 accolades, corresponding to an average of 49.17 citations per year. The 2020 papers by Sharma [54] and Sundarakani [55] have received 228 citations, respectively. This anthology of internationally acclaimed manuscripts highlights the scholastic influence and acknowledgment of these publications in numerous academic fields.

### 5.2. Co-Citation Analysis

Co-citation analysis is a commonly used bibliometric analysis tool that facilitates conceptual connections among influential articles within a specific subject, as well as the visualization of the conceptual framework of the area of study [1]. In a co-citation map, articles serve as nodes and the connections between them are depicted by edges. Visual representation highlights the co-occurrence of articles and emphasizes the relationships and connections among research papers. Each node on the map corresponds to a specific article, and the edges between nodes signify instances in which these articles are cited together in the literature. A co-citation map represents a comprehensive and insightful visualization of the interconnectedness of research within a specific area of study [2]. The co-citation network was derived using the following general formula:

Bcocitataion = A × C(1)

where A represents an article × C represents the cited reference matrix.

### 5.3. Cluster Analysis

Clustering serves as an additional method for enhancing bibliometric analysis aimed at generating thematic or social clusters based on the specific type of analysis undertaken. The process of grouping network clusters and monitoring their evolution is a significant approach to understanding the emergence and development of a field of study. Co-citation analysis and bibliographic coupling techniques reveal thematic clusters that shed light on the fundamental themes that influence the intellectual framework and their progression within the research field [56]. A range of clustering techniques such as exploratory factor analysis, hierarchical clustering, the island algorithm, the Louvain method, multidimensional scaling, and the simple center algorithm can be utilized to enhance the clustering process because they have the potential to mutually reinforce each other. Hierarchical clustering is widely recognized as the primary approach for categorizing data [44]. The extensive use of this tool is attributed to its adaptability and efficiency across multiple fields. A software application was employed to construct a graphical representation wherein distinct nodes within the network were highlighted and organized according to the provided inputs. An alternate approach involves the process of clustering the network’s nodes and generating a visual representation, or map, using specialized software to emphasize these clusters of nodes [56,57] 

Three primary research clusters were identified through cluster analysis to emphasize their respective research areas. The clusters identified according to the total link strength (TLS) are highlighted in Table 4 below.

In Cluster 1, the prominently identified articles were authored by Saberi et al. [58] with a total link strength (TLS) of 110. This study examines the associated articles on managing and controlling global food supply chains and food security aspects across difference regions globally. This study explores the potential of blockchain technology, which ensures transparency, traceability, and security, to mitigate issues in food traceability. This study critically examines the application of blockchain and smart contracts in supply chain management, driven by sustainability goals set by governments, communities, and consumers. The study conducted by Feng et al. [38] investigated the significance of traceability in food quality and safety management. This study highlights the importance of blockchain technology as an advanced solution for improving traceability by establishing security and transparency measures. This study introduces a framework for architectural design and an analysis flowchart for implementing food traceability systems based on blockchain technology. The findings of this study offer significant contributions toward enhancing the comprehension and efficacy of food traceability systems by employing blockchain technology. These insights provide crucial directions for researchers and practitioners to foster advancements in food sustainability. The research conducted by Behnke and Janssen [51] highlighted the importance of ingredient traceability in the face of growing global and complex food markets, emphasizing the necessity of meeting consumer demands for high-quality products. This study supports the use of blockchain technology to enhance traceability. They investigated four cases in the food supply chain through a template analysis of 16 interviews, revealing 18 boundary conditions categorized into business, regulation, quality, and traceability domains. The study by Kamble et al. [44] explores the transformative impact of blockchain technology in agriculture supply chains (ASC). This study focused on India’s agricultural sector and recognized the nation’s difficulties in guaranteeing food security for its growing population. This study identified and established relationships among 13 enablers of blockchain technology adoption in agriculture supply chains, emphasizing the significance of traceability, auditability, immutability, and provenance. This research unveiled a complex causal relationship among the identified enablers using a combined interpretive structural modelling (ISM) and Decision-Making Trial and Evaluation Laboratory (DEMATEL) methodology. The findings emphasize the potential of blockchain technology to streamline transactions, reduce intermediaries, and address payment delays. This study provides valuable insights for practitioners and policymakers in strategizing blockchain technology implementation in real-time, data-driven, and sustainable agricultural supply chains. Zhao et al. (2019) [59] highlight the significance of blockchain technology as a fundamental component of Industry 4.0 to guarantee data integrity, thwart manipulation, and eradicate single points of failure throughout the value chain. This study employs a comprehensive literature review to investigate the current state of blockchain technology and its recent advancements, applications, and challenges in the management of agri-food value chains. The study by Kamalakshi and Naganna [20] explores the integration of advanced technologies with blockchain to identify improvements in traceability, information security, manufacturing, and sustainable water management. It acknowledges obstacles, such as storage capacity, privacy concerns, cost, regulation, throughput, latency, and expertise. This study makes a scholarly contribution by revealing the potential of blockchain technology to improve the performance of the agri-food value chain in critical domains, such as food safety, quality, and traceability. The articles in Cluster 1 collectively underscore the capacity of blockchain technology to contribute to sustainability goals, mitigate complexities in supply chain management, and improve overall performance in critical domains such as food safety, quality, and traceability.

In Cluster 2, the study [52] with a TLS of 56 examined the escalating demand for efficient traceability throughout the agricultural and food supply chains, driven by heightened apprehensions regarding safety, quality, and validation standards. The proposal suggested utilizing the Ethereum blockchain and smart contracts as a means to augment traceability inside the soybean supply chain. This study suggests that smart contracts oversee the interactions between participants and record transactions in an unchangeable blockchain ledger. This study ensures transparency and traceability in a secure, trustworthy, and efficient manner. The proposed method eliminates the need for a centralized authority, thereby improving efficiency and safety and ensuring integrity and reliability. Francisco and Swanson [60] explore the role of blockchain in ensuring data integrity and preventing tampering in agri-food value chains by analyzing its applications in traceability, information security, manufacturing, and water management. This research contributes to the agri-food value chain literature by highlighting blockchain’s potential for enhancing food safety, quality, and traceability, while proposing future research directions to address identified challenges (Barbosa 2021 [24]). This study investigates the decentralized and transparent features of blockchain technology. This study utilized the Unified Theory of Acceptance and Use of Technology (UTAUT) paradigm to construct a conceptual model for supply chain traceability and highlighted the necessity for enhanced comprehension and implementation of blockchain technologies within the academic and managerial domains to fully harness their capabilities in enhancing transparency within supply chains. The scholarly publication by Nakamoto [3].) introduced a theoretical foundation for a decentralized electronic payment system that enables direct online transactions, thus obviating the need for intermediaries such as financial institutions. The proposed methodology involves implementing a decentralized network, wherein transactions are chronologically marked using a cryptographic proof-of-work mechanism, thus establishing an unalterable ledger. The proposed technique utilizes the longest blockchain to verify a sequence of transactions, thereby bolstering its resilience against future attacks by amassing significant processing power. Kamilaris et al. [61] explored the application of blockchain technology to decentralized and untrusted networks, thereby eliminating the need for intermediaries in transactions. The study analyzed several initiatives and discussed the consequences, obstacles, and potential benefits of their implementation. This study highlights the potential of blockchain technology to promote transparency across the food supply chain. This study offers a critical evaluation of the maturity levels exhibited by active projects, thereby providing valuable insights into the current status of blockchain applications within the agricultural industry. Lin et al. [62] addressed the growing concerns over food safety by emphasizing the need for an efficient traceability system to detect and prevent issues while ensuring accountability. Traditional systems encounter problems such as data invisibility and tampering. This study proposed a blockchain-based food safety traceability system that leverages characteristics such as irreversible time vectors, smart contracts, and consensus algorithms. A prototype system was developed, featuring management architecture for on-chain and off-chain data to address the blockchain data explosion issue in the IoT. An enterprise-level smart contract was designed to prevent data tampering and the sensitive disclosure of information. The Cluster 2 publications examined in this study mainly focus on utilizing blockchain technology to enhance traceability, transparency, and security within various supply chains. These articles discussed the use of blockchain technology, smart contracts, and decentralized networks to eliminate the need for centralized governing bodies. This approach ensures transaction security, reliability, and efficiency. Table 4 presents the lead papers in all clusters.

**Figure 9:**
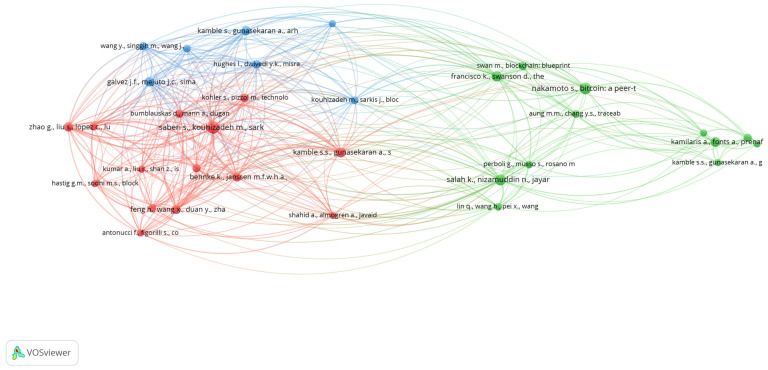
Co-citation analysis of clusters [three clusters emerged as green, blue, and red themes] [source: authors’ own work].

In Cluster 3, Galvez et al. [63] examined the issue of food falsification, which resulted in significant economic losses. This study proposed the use of blockchain technology to augment traceability and transparency within the food supply chain. Blockchain was introduced as an innovative and crucial approach to ensure the integrity of the analytical process, particularly regarding the accumulation and maintenance of data. This study considered blockchain technology a feasible method for locating and mitigating contamination sources throughout the global food supply chain. Hughes et al [64] used an IS/IM lens to conduct a comprehensive review and identify potential blockchain applications. This study explores key themes, articulating numerous possible applications and the future direction of blockchain technology. Kamble et al. [44] suggested that blockchain has the potential to contribute to the United Nations’ sustainable development goals and bring about extensive changes in established industries and practices. This study explores the transformative potential of blockchain technology in supply chains to enhance visibility and transparency while addressing trust-related issues. This study introduced a model based on technology acceptance, readiness, and planned behavior theories to understand user perceptions of blockchain technology adoption. The model was statistically validated through a survey of 181 supply chain practitioners in India. Practitioners considered blockchain technology adoption uncomplicated, which confers significant advantages in terms of enhancing supply chain efficacy. Kouhizadeh and Sarkis [65] recognized blockchain technology as an early-stage and currently prevalent innovation, with the primary aim of stimulating discussion and encouraging further practice and research at the intersection of green supply chains and blockchain technology. This study offers a comprehensive analysis of the fundamental aspects of blockchain technology, elucidates several practical applications and challenges, and proposes potential avenues for future research. Abeyratne and Monfared [22] examined the use of blockchain technology as a fundamental framework for distributed ledgers, offering a decentralized and transparent system for conducting transactions. The inherent characteristics of blockchain enhance trust through transparency and traceability in various transactions. This study provides a comprehensive analysis of the existing state and practical implementation of blockchain technology, focusing on its potential advantages within the context of manufacturing supply chains. The authors provided a conceptual framework for implementing a blockchain-enabled manufacturing supply chain, illustrating their argument through a cardboard box production case study within a worldwide network. This study discusses the prerequisites and potential obstacles to integrating blockchain technology into forthcoming manufacturing systems. Figure 9 presents the results of the co-citation analysis.

**Figure 10:**
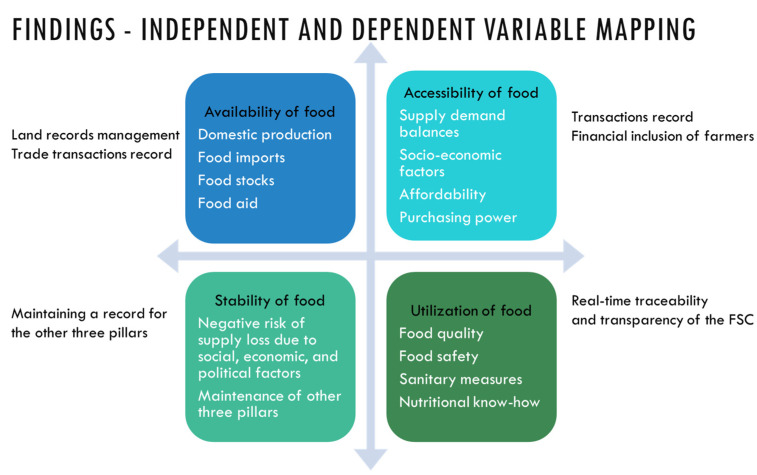
Findings—independent and dependent variable mapping [source: authors’ own work].

Based on the outcome of the clusters that emerged in the co-citation analysis, this research then draws mapping of these into the four dimensions of the food security pillars as shown in Figure 10. This mapping leads to identifying variables and sub-variables that lead to a dependent variable that measures the food security performance outcome [66]. The outer determinants are the factors that drive the blockchain associations and impact the four dimensions of food security. This framework development could be tested and validated by researchers when implementing the framework in their agri-food supply chain projects and food security initiatives. Academic researchers can further test this framework based on empirical research in their own geographical countries and industry verticals.

## 6. Conclusions

This study conducted a bibliometric analysis to investigate the impact of blockchain technology on guaranteeing food security in the UAE. In total, 489 articles obtained from the Scopus database were examined. Using bibliometric analysis methods and methodologies, this study identified prominent journals, influential institutions, and notable papers of substantial influence that are now gaining popularity in blockchain technology and food security within the UAE. Based on the number of articles, the three prominent sources are *Sustainability* (Switzerland), *IEEE Access*, and *the Journal of Cleaner Production*. *Sustainability* (Switzerland) is the leading publication with 28 articles, emphasizing its substantial contribution to the field. *IEEE Access* holds a notable position with 24 articles, highlighting its significance in disseminating research across various domains. *The Journal of Cleaner Production* ranks third with 22 articles, highlighting its commitment to promoting environmentally friendly practices and sustainable production methodologies. Collectively, these sources play a pivotal role in shaping the discourse and advancing knowledge within their respective domains, reflecting the diverse landscapes of scholarly research on sustainability, technology, and clean production. The articles authored by Saberi et al. [58], Salah et al. [52], and Galvez et al. [63] were the most recent and significant developments after the implementation of co-citation analysis. The analysis successfully identified prominent themes in these articles and laid the groundwork for defining potential avenues for future research in these disciplines.

### Future Research

For future research, it is crucial to thoroughly explore the practical application of blockchain technology in the UAE’s food supply chain. It is essential to examine real-life examples and evaluate the capacity for the growth and flexibility of blockchain applications. Furthermore, it is worth investigating the amalgamation of developing technologies, such as the Internet of Things and artificial intelligence with blockchain to achieve a more complete strategy for ensuring food security. Furthermore, it is crucial to carefully assess the legislative and policy ramifications of introducing blockchain technology in the UAE’s food sector to guarantee a smooth and enduring shift. As technology advances, ongoing research and innovation in this field will become crucial in determining the future of food security plans in the UAE.

## Figures and Tables

**Table 1:** Bibliometric analysis results by journal [source: authors’ own work].

Sources	Articles
*Sustainability* (Switzerland)	28
*IEEE Access*	24
*Journal Of Cleaner Production*	22
*Foods*	11
*Sensors*	11
*Computers and Electronics in Agriculture*	7
*International Journal of Advanced Computer Science and Applications*	7
*Operations Management Research*	6
*Agriculture* (Switzerland)	5
*IEEE Transactions on Engineering Management*	5

**Table 2:** Keyword occurrences [source: authors’ own work].

Words	Occurrences
blockchain	269
block-chain	163
food supply	137
supply chains	116
internet of things	111
agriculture	105
supply chain management	75

**Table 3:** The most highly cited literature in the field [source: authors’ own work].

Source	Total Citations	TC per Year
Dutta P, (2020) [49]	469	117.25
Kamble S (2021) [44]	463	115.75
Mistry I, (2020) [50]	385	96.25
Salah et al. (2019) [52]	372	74.4
Behnke and Janssen (2020) [51]	364	91.00
Leng K, (2020) [53]	295	49.17
Sharma et al. (2020) [54]	293	73.25
Sundarakani et al. (2022) [55]	228	70.5

**Table 4:** Lead clusters as emerging from the research [source: authors’ own work].

Cluster 1	Total Link Strength
Saberi et al. (2019) [58]	110
Feng et al. (2020) [38]	79
Behnke and Janssen (2020) [51]	72
Kamble et al. (2020) [44]	69
Zhao et al. (2019) [59]	69
**Cluster 2**	**Total Link Strength**
Salah et al. (2019) [52]	56
Francisco and Swanson (2018) [60]	46
Nakamoto, n.d. [3]	36
Kamilaris et al. (2019) [61]	35
Lin et al. (2019) [62]	32
**Cluster 3**	**Total Link Strength**
Galvez et al. (2018) [63]	83
Hughes et al. (2019) [64]	61
Kamble et al. (2021) [44]	59
Kouhizadeh and Sarkis (2018) [65]	49
Abeyratne and Monfared (2016) [22]	34

## Data Availability

The original contributions presented in the study are included in the article, further inquiries can be directed to the corresponding author.
